# Phase Variation in *Myxococcus xanthus* Yields Cells Specialized for Iron Sequestration

**DOI:** 10.1371/journal.pone.0095189

**Published:** 2014-04-14

**Authors:** Katarzyna Dziewanowska, Matthew Settles, Samuel Hunter, Ingrid Linquist, Faye Schilkey, Patricia L. Hartzell

**Affiliations:** 1 Department of Biological Sciences, University of Idaho, Moscow, Idaho, United States of America; 2 The Institute for Bioinformatics and Evolutionary Science, University of Idaho, Moscow, Idaho, United States of America; 3 Bioinformatics and Computational Biology, University of Idaho, Moscow, Idaho, United States of America; 4 National Center for Genome Resources, Santa Fe, New Mexico, United States of America; University of Strathclyde, United Kingdom

## Abstract

*Myxococcus xanthus* undergoes phase variation during growth to produce predominantly two colony phenotypes. The majority are yellow colonies containing swarm-proficient cells and a minority are tan colonies containing swarm-deficient cells. Comparison of the transcriptomes of a yellow variant, a tan variant, and three tan mutants led to the identification of differentially-regulated genes that define key segments of the phase variation pathway. For example, expression of genes for the yellow pigment DKxanthene and the antibiotic myxovirescin was increased significantly in yellow variants. In contrast, expression of the siderophore myxochelin, hemin binding proteins, and iron transport proteins was increased specifically in tan strains. Thus, a consequence of phase variation is that yellow cells shift from producing antibiotic and pigment to producing components involved in acquisition of iron, which may increase fitness during periods of iron limitation. Multiple protein kinases and HTH-Xre DNA-binding proteins identified in this study may be involved in the regulatory hierarchy that governs phase variation.

## Introduction

Phase variation (PV) is a form of phenotypic plasticity that allows a single bacterial species to persist in alternate forms by expressing different sets of genes. Organisms that undergo phase variation alter the expression of various cellular components via a genetic [1] or epigenetic [2], [3] switch. Typically, the alternative programs result in production of different combinations of proteins, lipids, or carbohydrates on the cell surface that contribute to phase-specific changes in colony texture, color, or morphology [4].

Pathogenic strains that undergo PV produce cell types that are less likely to be recognized by the immune system, thus affording these pathogens enhanced opportunities to survive in the host. While human pathogenic strains have been the subject of intense research, non-human pathogens similarly benefit from phase switching. *Photorhabdus luminescens* produces M and P- morphotypes during phase variation. The P-form cells, produce larger colonies, more secondary metabolites, and generate more bioluminescence than the smaller M-form cells. The M form is critical for the life cycle of *P. luminescens* because it is the form that can persist in the nematode host *Heterorhabditis bacteriophora* [5].

*M. xanthus* is a nonpathogenic bacterium with a biphasic life cycle comprised of a vegetative phase and a complex developmental phase that together ensure survival in harsh environments. Multiple studies have documented PV in *M. xanthus* and its effects on vegetative growth, swarming, and development [6]–[10]. The predominant morphotype, or variant, in *M. xanthus* produces a rough, matte colony that is yellow due to production of the polyketide pigment DKxanthene (DKX). Yellow variants are proficient at swarming on agar surfaces. Colonies of the other morphotype are tan and generally have a smooth, shiny surface. Tan variants exhibit reduced swarming. The *P. luminescens* M and P-forms share features in common with the yellow and tan variants of *M. xanthus* including a property where the one form - the tan variant- can be ingested by bacteriophagous nematodes, while the other form – the yellow variant - is resistant to the nematode [10]. The ability to produce a variant that is resistant to a predator may enhance survival of *M. xanthus* in its soil habitat.

Yellow and tan colonies do not represent pure populations of either variant type, but rather are dynamic mixtures. Yellow colonies contain cells capable of giving rise to ≈95% yellow (range 75–99%) colonies and 5% tan colonies (range 1–25%); these ratios are reversed in the tan variants. The rate of switching from yellow to tan has been reported to be as high as 10^−2^ to 10^−3^ per cell per generation; switching from tan to yellow is higher, so colonies of the yellow variant outnumber those of the tan variant [11].

*M. xanthus* cells aggregate together to form fruiting bodies filled with heat and desiccation-resistant spores in response to starvation [12]. Cultures that are predominantly yellow form dark fruiting bodies within which roughly 10% of the cells differentiate into spores. Tan variants typically form immature mounds that are light in color because fewer than 0.1% of the initial cell population differentiates into spores. Although the tan variants produce few spores when developed on their own, they contribute disproportionately to the population of heat-resistant spores when allowed to develop in the presence of yellow variants [6], [11]. This suggests that yellow cells provide some factor that the tan cells need to produce viable spores. One factor needed by tan variants is DKX, the secondary metabolite that confers the characteristic color of the yellow variants [13]. Strains with mutations in the *dkx* biosynthetic operon are tan in color and are deficient in their ability to produce heat-resistant spores. Addition of purified DKX partially rescued the sporulation defect of the *dkx* mutants [13].

Here we compare the transcriptome of the wild-type tan variant (WT-T) with that of three tan mutants. We identified >200 genes whose expression was increased or decreased at least 7.3 fold in two or more of the strains and found multiple components that can serve as biomarkers for PV. Despite their distinct genetic backgrounds, all four tan strains showed significant increases in expression of genes whose products are needed for acquisition of iron and most showed significant decreases in expression of genes for synthesis of DKX and an antibiotic named myxovirescin (also known as TA). Bioassays confirmed that changes in transcription correlated with changes in the amount of DKX, myxovirescin, and the siderophore myxochelin. Sets of genes encoding serine-threonine protein kinases and helix-turn-helix xenobiotic response elements (HTH-Xre) were up-regulated in tan variants. Results suggest that some of these genes act downstream of the HTH-Xre228 protein, which previously was found to regulate expression of *dkx* genes [9].

## Results

### Phase variation affects multiple phenotypes

Phase variation in *M. xanthus* is pleiotropic: phenotypes including pigment and antibiotic production, swarming, and development are affected. To identify genes and regulatory processes that contribute to these phenotypes, the transcriptomes and behaviors of four tan strains were compared with the yellow WT parent (WT-Y). The tan strains included the wild-type tan variant (WT-T) and three mutants. Two of the tan mutants, *xre228* (*MXAN_0228*) and *asgB* (*MXAN_2913*), were chosen because they carry mutations in putative or known regulatory genes. The *xre0228* gene encodes a putative member of the HTH-XRE superfamily that is differentially regulated in yellow and tan variants [9]. The *asgB* gene encodes a 163 amino acid DNA binding protein whose C-terminal region shares similarity with region 4 of the sigma 70 protein family and the HTH-XRE superfamily [14]. The mutant has a profound developmental defect [15]. The *asgB* mutant constructed for this study was initially yellow but within a few weeks, it was tan and did not revert to yellow. The third tan mutant, *dkxG* (*MXAN_4299::Himar*), was chosen as it carries a transposon mutation in a gene that encodes a polyketide synthase required for DKX biosynthesis [9]. The *dkxG* mutant is tan due to loss of pigment.

Cell cohesion (aka agglutination, aggregation) has been used to differentiate strains on the basis of surface components, particularly fibrils [16], [17]. Loss of fibril material occurs in a subset of mutants, such as *dsp/dif*, that are defective in social gliding [18]. We compared the cohesive properties of WT-Y against the four tan strains. The WT-Y strain was proficient for cohesion and precipitated from solution (Fig S1). Two of the tan mutants, *dkx* and *xre228* aggregated as well as or better than the WT-Y, respectively. In contrast, the WT-T and *asgB* mutant strains remained suspended with little change in optical density associated with settling of aggregated cells (Fig S1).

A hallmark of *M. xanthus* is its ability to form spore-filled multicellular fruiting bodies in nature. In the laboratory, this complex development cycle can be induced and synchronized by removing nutrients from a growing population of cells and simultaneously concentrating the cells to achieve a high cell density [19]. Development can ensue when concentrated, starved cells are placed on a solid surface devoid of nutrients. The WT-Y strain formed dense mounds after 48 hr whereas WT-T formed only soft mounds or ridges. With additional time some of the mounds produced by WT-T cultures darkened slightly but never achieved the dark, dense fruits of the WT-Y. The *dkx* and *asgB* tan mutants showed significant delays in fruiting or failed to develop (data not shown, [13], [14]. The *xre228* mutant produced near normal fruiting bodies, but the number of viable heat-resistant spores was reduced [9].

### Expression of myxochelin and iron acquisition systems is increased in tan variants and mutants

**Figure 1:**
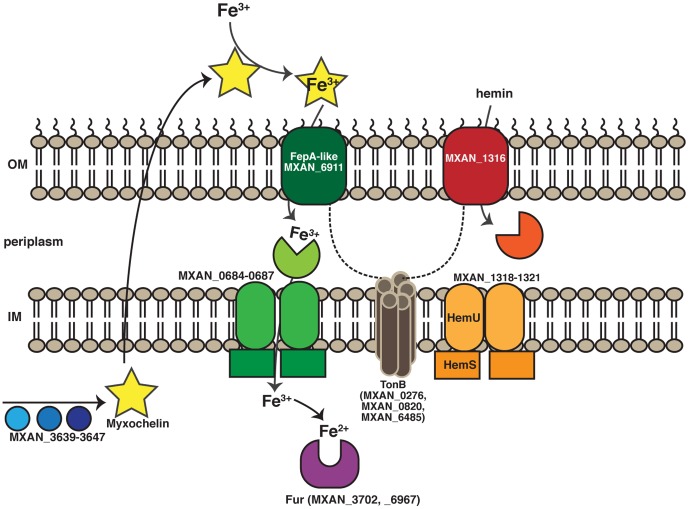
Tan strains activate pathways dedicated to acquisition of iron. Many of the genes whose expression is increased significantly in the tan strains comprise the siderophore production and transport, hemin transport, and proteins that provide energy for transport of iron-binding compounds. A partial list of components produced in tan cells is shown in this cartoon. The blue/purple circles represent the siderophore myxochelin pathway (MXAN_3639-3647) [39], [40]. Myxochelin (*mxc*) is shown as a yellow star that binds Fe^3+^ when exported. Mxc•Fe^3+^ may be imported by a FepA-like protein (candidate is MXAN_6911; dark green shape), located in the outer membrane [41]. Light green shapes represent the ferric siderophore ABC transporter genes (permease and MXAN_0684-0687 in membrane); brown cylinders represent periplasmic energy transduction TonB proteins (MXAN_0276, MXAN0820, and 6485) that may energize the outer membrane proteins (dashed lines). Red/orange icons represent hemin transporters (MXAN_1318-1321). Not shown are RhtX/FptX siderophore transporter (MXAN_5357), iron ABC transporters (MXAN_0770-0772), iron compound ABC transporter, and a periplasmic iron compound binding protein, Fe•ABC (MXAN_6000). Search Tool for the Retrieval of Interacting Genes/Proteins (STRING) software [42] predicts MXAN_3639 (putative iron-chelator utilization protein in the myxochelin operon) interacts with the ferric siderophore ABC transporter permease (MXAN_0685, 0686).

The expression profiles of the WT-T and tan mutants were compared to the WT-Y reference strain variant using a custom DNA tiling array and RT-PCR. These data are summarized in Table 1; data for individual genes are listed in Table S1. Expression of genes involved in acquisition and transport of iron was increased significantly in tan strains. Fig 1 shows the likely relationship between the products of these genes. Expression of all six genes of the regulon encoding hemin transport proteins (*MXAN_1316-1321*) was increased >5-fold in all four strains. The nine genes of the myxochelin siderophore biosynthesis pathway (*mxc*, MXAN_3639-3647), which share homology with the genes characterized for myxochelin synthesis and transport from *Sorangium* [20] increased 5 to 55-fold.

**Table 1:** Expression of genes for iron acquisition is increased in tan strains.

		Fold change* in			
MXAN ID	Description	WT-T	*asgB*	*dkxG*	*xre228*
1316 to 1321	Genes for hemin transport system	+6 to +13	+8 to +43	+6 to +16	+3 to +6
1688 to 1690	TonB family protein, CHP, esterase	+6	+20	+16	+5
3639 to 3647	Genes for myxochelin biosynthesis (*mxc*)	+8 to +55	+15 to +46	+18 to +48	+8 to +17
4198 to 4201	Genes for putative OM macrolide efflux, ABC protein family	+75 to +119	+15 to +35	+216 to +412	+60 to +137
6000	Fe•ABC periplasmic iron compound-binding transporter	+10	+17	+10	+7
6911	TonB-dependent receptor	+61	+278	+39	+6

Legend: * for multigene clusters, the range of expression change values is given.

Partial list of genes showing significant expression changes in the *M. xanthus* tan variant and three tan mutants (12 datasets: four biological replicates with triplicate technical replicates) in DNA tiling array studies relative to the WT-Y variant (data not shown). Expression patterns consistent in four different tan *M. xanthus* strains (data for individual genes are provided in Table S1). MXAN ID and Description refers to annotation; both are derived from the *M. xanthus* genome [43]; fold change represents the increase (A) or decrease (B) in expression and is derived from coefficient value.

Many Gram-negative bacteria produce a protein named Fur (ferric uptake regulator) that uses iron (Fe^2+^) as a cofactor to regulate expression of genes involved in production of siderophores and even toxins [21]. Expression of two Fur-like homologs, MXAN_3702 and MXAN_6967 that share 36% (47/129) and 30% (37/125) identity with the *E. coli* Fur protein (EHU14036) respectively was increased in tan variants. Membrane components involved in transport of siderophores, including a putative Fe-ABC periplasmic iron compound binding protein transporter, a FepA homolog (TonB-dependent outer membrane receptor), and a TonB-family protein that likely energizes an outer membrane receptor were increased in tan strains.

### Siderophore production is increased in tan strains

The results above showed that expression of genes whose products are needed for iron acquisition is increased in tan strains. To determine if increases in transcription correlated with actual changes in production of iron-binding components, levels of siderophore in the WT-Y, WT-T, and mutant strains were assayed. *M. xanthus* produces a catecholate type of siderophore named myxochelin [20] which was assayed using chrome azurol S (CAS) [22]. When aliquots of 10^7^ cells were spotted on CAS-Fe agar medium, tan *M. xanthus* strains produced an orange halo that was 2-fold greater (range 1.8 to 2.4-fold increase, n = 8) than that of a yellow strain (Fig S2). The orange halo is produced because the high affinity siderophore secreted by cells removes iron from the CAS-Fe complex, resulting in conversion of the blue CAS-Fe color to the orange CAS color. This plate assay uses a CAS-Fe complex containing the detergent hexadecyltrimethylammonium bromide (HDTMA) to observe production of a halo around individual colonies as they grow.

To ensure that the difference in the siderophore zone was not an artifact caused by increased sensitivity of tan strains to the detergent, we compared the level of siderophore in the spent medium (supernatant) and cell extract of broth-grown cultures. The siderophore units were calculated from the absorbance at 630 nm of cell-free supernatants of WT-Y, WT-T and mutant cultures incubated with CAS-Fe solution [22]. To facilitate direct comparison, samples were harvested when the cultures were at the same cell density (≈5×10^8^ cells ml^−1^; mid-log phase of growth).

**Figure 2:**
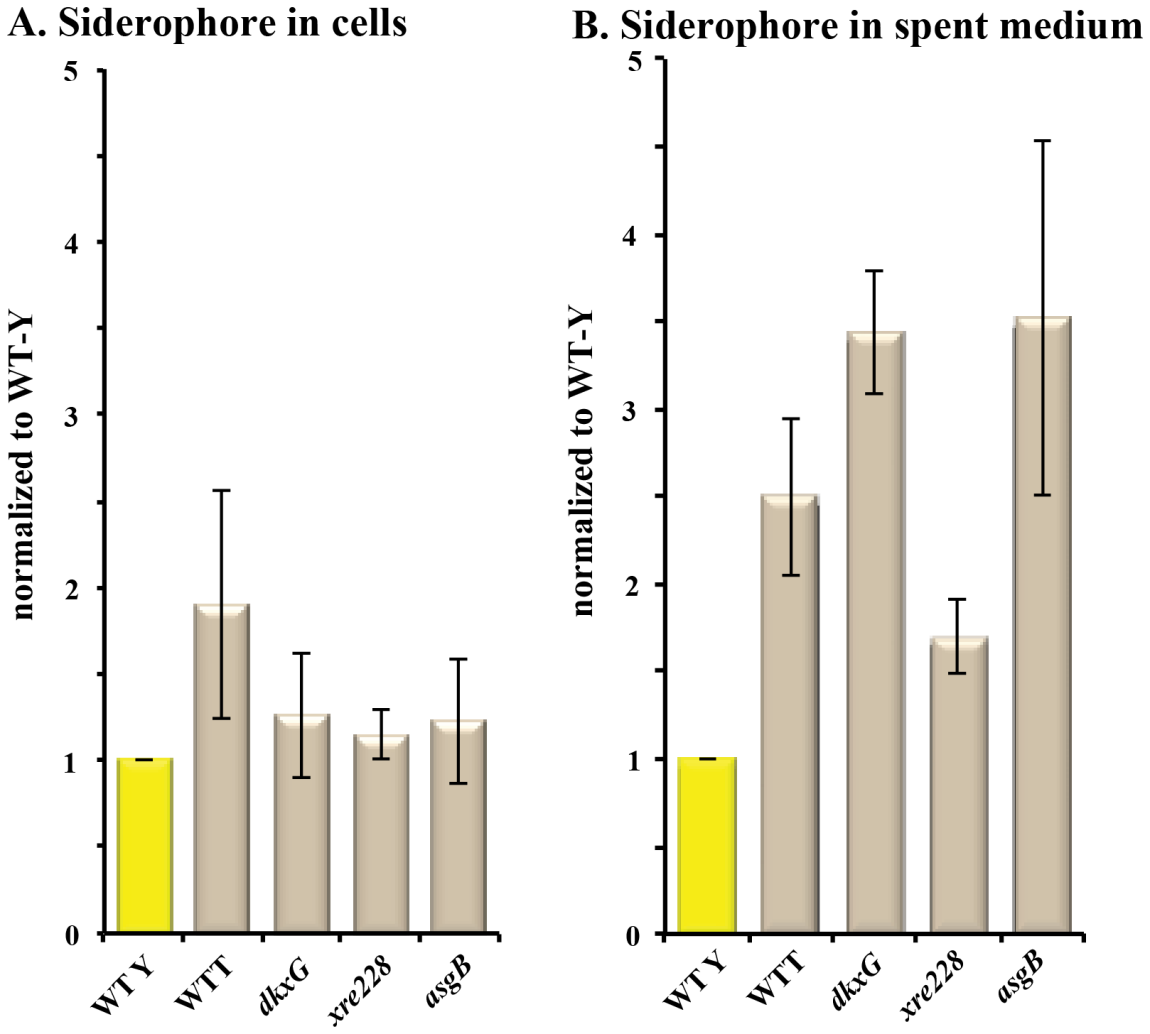
Tan mutants produce and secrete more siderophore. Chrome azurol S assays were performed as described in Methods. Yellow bars signify the yellow strain; tan bars signify tan strains. (A) The amount of siderophore in 2×10^8^ whole cells after lysis is reported as siderophore units relative to the WT-Y strain (100%). (B) Culture supernate (spent medium) was taken when the density was at ≈5×10^8^ cells ml^−1^ and was used to quantify the amount of released siderophore. Samples represent the average of three independent assays.

All of the tan strains secreted more siderophore than the WT-Y strain. As shown in Fig 2B, the amount of siderophore detected in the spent medium of the tan strains increased 175% to 350% compared with the WT-Y strain. The abundance of siderophore in the supernatant was not due to leakage of tan strains because the amount of siderophore in the cell pellet, lysed in 1% NP-40 detergent to release bound siderophore, was similar in the tan strains and the WT-Y (Fig 2A). The increased amount of siderophore released by tan strains is consistent with the increased expression of myxochelin genes.

### Iron plays a role in regulation of DKX and siderophore

The increase in iron acquisition components in tan cells suggested that phase variation may be a response to iron limitation. To explore the role of iron in phase variation, the effects of iron availability on levels of the PV biomarkers DKX and siderophore were compared. WT-Y and WT-T cells were inoculated into separate batches of rich medium (casitone Tris, phosphate, Mg^2+^; CTPM) and split into four 12 ml batches, which were supplemented with H_2_O, FeCl_3_, dipyridyl, and CuCl_2_. Aliquots (5 ml) from each batch were transferred to Klett flasks and incubated at 32°C with shaking. Cells and supernates were harvested when the density reached 6×10^8^ cells ml^−1^.

**Figure 3:**
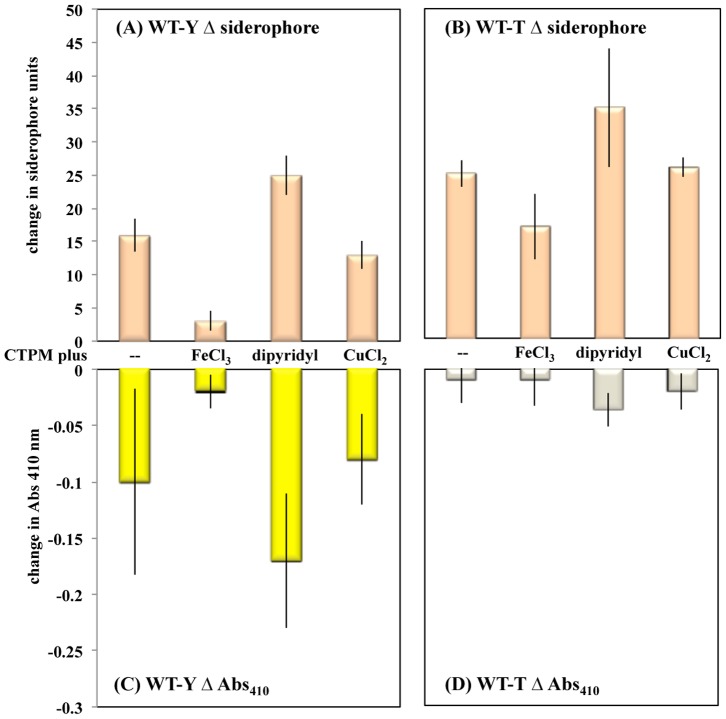
Effect of iron on production of PV molecules. Parallel cultures of WT-Y and WT-T strains were grown with and without supplements as described in Methods; siderophore and Abs_410_ were assayed when cells reached 6×10^8^ cells ml^−1^. Data represent the average of duplicate readings obtained after performing three independent tests comparing the endpoint against the cells used as inoculate. (A) change in siderophore WT-Y, (B) change in siderophore WT-T, (C) change in Abs _410_ WT-Y, and (D) change in Abs _410_ WT-T.

Fig 3 shows that siderophore levels increased and relative DKX levels (estimated from Abs_410_) decreased in all samples compared with those from the inoculum (4×10^8^ cell ml^−1^). The changes detected in WT-Y cells growing in CTPM are consistent with a decrease in the ratio of yellow to tan cells in a WT population as it ages. Siderophore (Fig 3A) and DKX (Fig 3C) levels were stable in the presence of FeCl_3_ compared with the CTPM control. Accumulation of fewer tan variant cells in the population could account for the smaller changes in siderophore and DKX. In contrast, dipyridyl stimulated siderophore production 8X and decreased the relative DKX value 8.5X compared with FeCl_3_. CuCl_2_ did not have the same effect as FeCl_3_, which shows that the response is specific to iron.

The response of WT-T cells to different concentrations of iron in growth medium followed the same trend as WT-Y, but was less dramatic. Levels of siderophore are elevated in tan variants compared with yellow variants and added iron resulted in only a small decrease compared with the CTPM control (Fig 3B). As expected, the change in DKX (Fig 3D) was minimal. Similar results were seen on solid medium. Colonies of WT-Y on plates containing 0.5 mM FeCl_3_ remained yellow for more than a week, while colonies on standard medium began to turn brown after the same time period (data not shown). Brown coloration is thought to be due to secretion of the myxochelin siderophore and other compounds.

The results suggest that (1) the switch from yellow to tan variant is delayed significantly by high levels of iron and accelerated by removal of iron and (2) iron does not promote a switch from tan to yellow. With regard to the latter, WT-T cells may have a reduced ability to sense iron.

### Addition of iron suppresses the yellow to tan conversion

To test the hypothesis that high levels of iron impede the yellow to tan switch, we assayed the number of yellow and tan colonies recovered on plates after first growing WT-Y cells in medium with and without added iron. WT-Y cells, grown to the same density (5×10^8^ ml^−1^) in CTPM and CTPM+1 mM FeCl_3_ yielded 94% (n = 341) and 98% (n = 449) yellow colonies respectively when plated on standard CTPM medium. When the iron-grown cells were plated on CTPM plus FeCl_3_, 100% of the colonies that arose were yellow (n = 312). In contrast, when cells were plated on CTPM with 25 µM dipyridyl, 59% of the colonies were yellow (n = 380).

To rule out the possibility that dipyridyl-induced lethality of yellow cells was skewing results, a parallel experiment was performed using mixtures of marked and unmarked cells. Equal numbers of Kan^R^ tan and Kan^S^ yellow variants were mixed and plated on CTPM medium with 25 µM dipyridyl. After 6 days, tan colonies (n = 69) were replica-plated to CTPM and CTPM+Kan media. 100% of colonies (n = 69) grew on CTPM medium; 31 of these also grew on CTPM+Kan. Hence more than half (38/69) of the colonies originated from the yellow WT parent. Indeed, after transfer from CTPM+dipyridyl back to CTPM, 92% of the recovered Kan^S^ colonies (35/38) were yellow. This shows that dipyridyl does not selectively kill yellow variants.

To determine if other elements could elicit the same response as iron in preventing the yellow to tan switch, an add-back experiment was performed. WT-Y cells were plated in parallel on CTPM medium with 25 µM dipyridyl plus (a) no addition, (b) 200 µM FeCl_3_, (c) 200 µM CuCl_2_, and (d) 200 µM ZnCl_2_. We found (a) 41% yellow (88/210), (b) 78% yellow (147/188), (c) 56% yellow (149/267) and (d) 49% yellow (114/233). Hence, addition of FeCl_3_ largely rescued or prevented the yellow to tan switch in the population; copper and zinc provided some protection.

### Iron represses expression of genes encoding myxochelin biosynthetic proteins and a FepA-like protein

**Figure 4:**
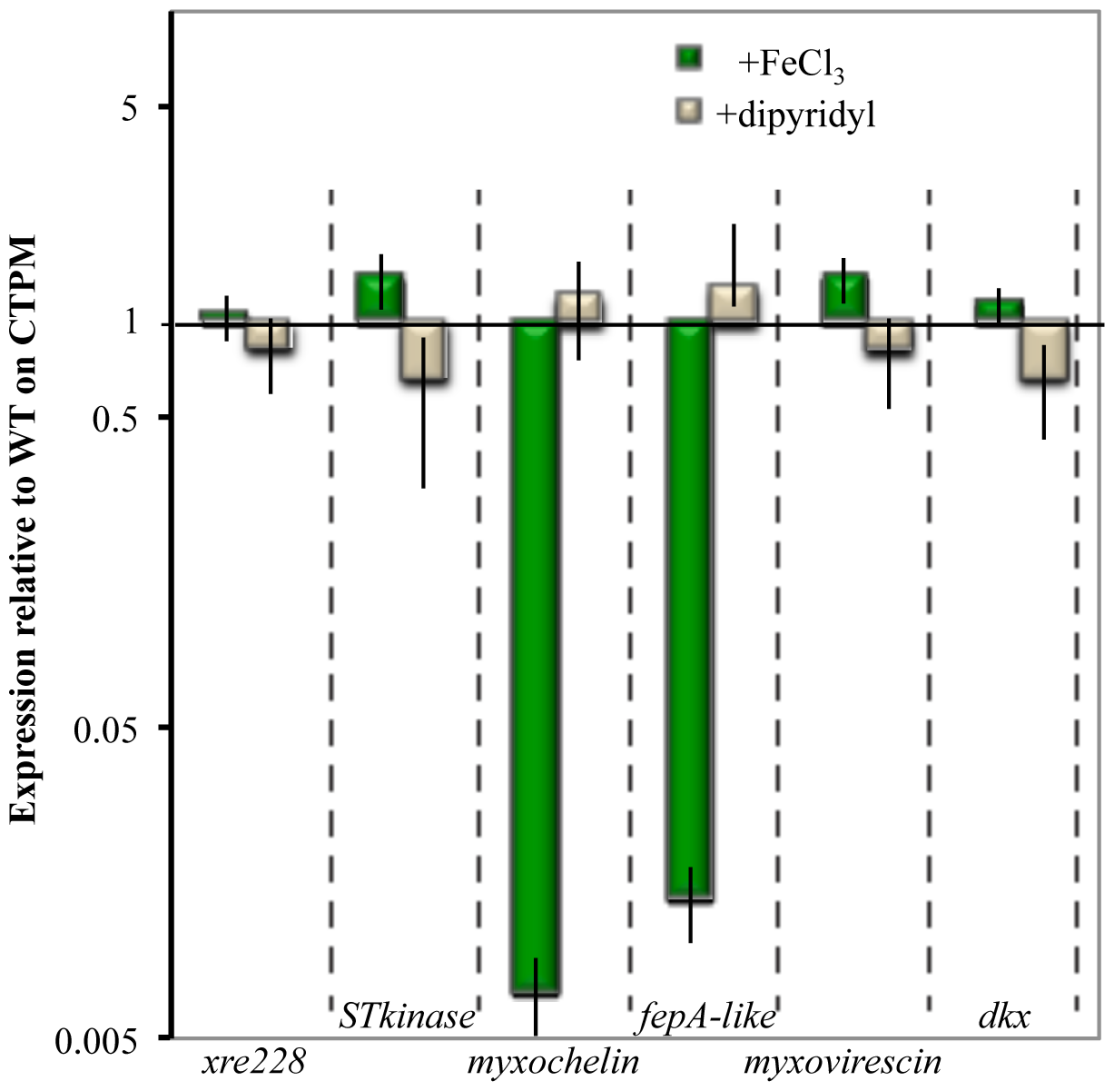
Regulation of gene expression by iron. RNA harvested from WT-Y cells was used to prepare cDNA for qRT-PCR analysis of representative genes whose expression is affected during phase variation; *MXAN_0228* (Xre228), *MXAN_7370* (ST_kinase), *MXAN_3641* (myxochelin), *MXAN_6911* (FepA homolog), *MXAN_3639* (myxovirescin) and *MXAN_4305* (DKX). The results represent three biological and technical replicates; data are from cells grown in CTPM+FeCl_3_ and CTPM+dipyridyl medium relative to levels from cells grown in CTPM alone (value  = 1) are presented.

The results shown in Fig 3 showed that different concentrations of iron can affect DKX and siderophore production. To determine if similar changes would affect gene expression, we used RT-PCR to compare the expression of a subset of genes from WT-Y cells grown on CTPM, CTPM+FeCl_3_ and CTPM+dipyridyl. Fig 4 shows the relative changes in expression of genes in cells grown in CTPM+iron and CTPM+dipyridyl (reduced iron) relative to the CTPM control. Expression of DKX, myxovirescin (Mxv), and ST-kinase (*MXAN_7370*) was increased in the presence of iron and decreased when iron was removed. The opposite was true for *MXAN_3641* (myxochelin) and *MXAN_6911* (FepA homolog), both of which are involved in iron acquisition.

Data from independent experiments presented thus far show that iron influences gene expression and production of compounds that are linked to PV in *M. xanthus*. With regard to gene expression and phenotype, depletion of iron is sufficient to phenocopy WT-T in an otherwise WT-Y strain.

### Production of DKxanthene and myxovirescin (antibiotic TA) is decreased in tan mutants

The species name for *M. xanthus* is from the Latin word for yellow, the color of the compound DKxanthene. Loss of pigment in the tan strains was due to a significant decrease in expression of *dkx* genes (*MXAN_4289-4305*) in WT-T [9], and the *asgB* and *xre228* mutants (Table 2 and Table S1). Expression of the operon containing *dkxG* is decreased in the tan *dkxG* mutant, but expression of genes in other *dkx* operons was not affected.

**Table 2:** Expression of DKxanthene and myxovirescin (antibiotic TA) is decreased in tan strains.

		Fold change* in			
MXAN ID	Description	WT-T	*asgB*	*dkxG*	*xre228*
3930 to 3950	Genes for Mxv synthesis	−5 to −208	−6 to −195	−1 to −6	−5 to −176
4289 to 4305	Genes for DKX synthesis	−19 to −316	−21 to −584	−1 to −59	−28 to −295

*For multigene clusters, the range of expression change values is given.

Legend: See Table 1 legend.

**Figure 5:**
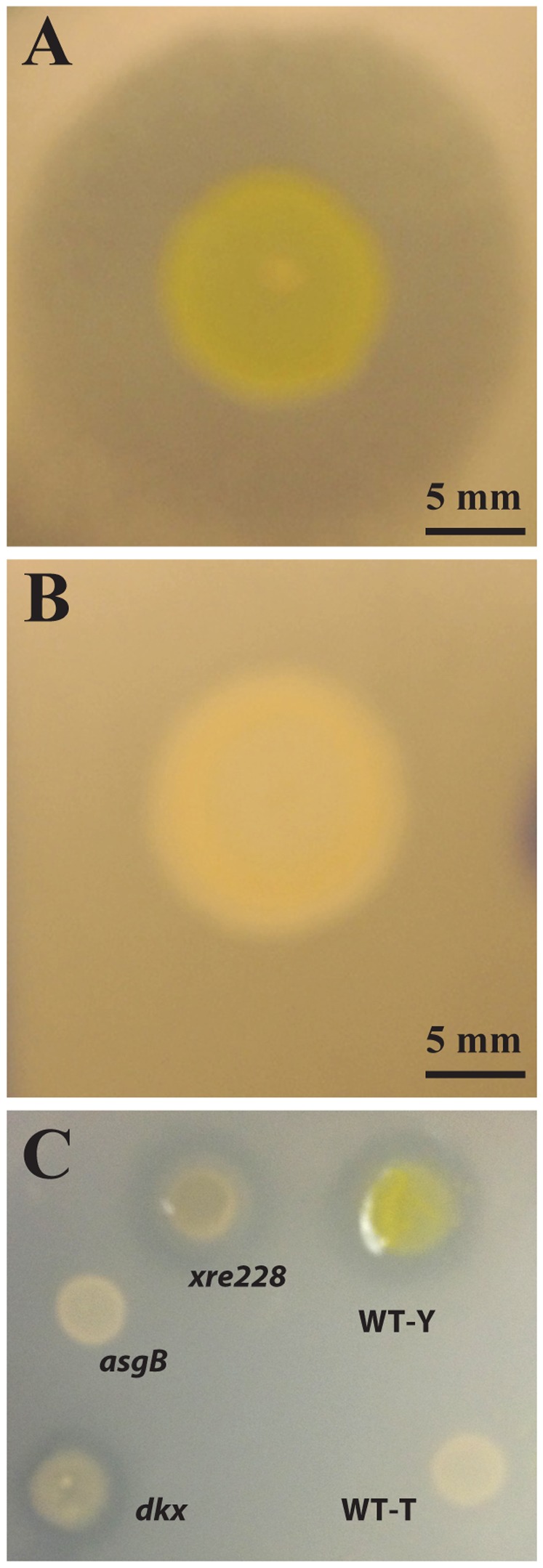
WT-T strains do not produce myxovirescin. Production of myxovirescin, also known as antibiotic TA, was monitored using a zone of inhibition assay with *E. coli* DH5α. Myxovirescin was produced by WT-Y variants (panel A), but not WT-T variant (B). (C) Like WT-T, myxovirescin was absent in *asgB*, but present in *dkxG* and *xre228* mutants. Production was delayed in *xre228*.

Yellow variants secrete a secondary metabolite called myxovirescin, an inhibitor of type II signal peptidase, to prey on other organisms [23], [24]. Expression of genes involved in synthesis of myxovirescin (*mxv*; *MXAN_3930-3950*)(Table 2) was decreased significantly in the WT-T, *xre228*, and *asgB* strains but decreased only slightly in the *dkxG* mutant. Mxv can be detected using a bioassay that measures inhibition of *E. coli* growth [24]. A zone of inhibition, due to secretion of Mxv, was detected for WT-Y (Fig 5A), but was absent in the WT-T variant (Fig 5B), consistent with the transcriptome data. Similarly, as shown in Fig. 5C, the bioassay confirmed the loss of Mxv in the *asgB* mutant and presence of Mxv in the *dkxG* mutant. Surprisingly, although we did not detect *mxv* transcript in the *xre228* mutant, the mutant produced a zone of inhibition (Fig 5C). The zone of clearing was visible around the WT-Y strain 18 hr after overlaying with *E. coli* while the zone of clearing around the *xre228* mutant was visible only after 30 hrs. This suggests that expression of *mxv* may be delayed in the *xre228* mutant or is induced by contact with a solid surface, high cell density, or by exposure to *E. coli*. Delayed expression would explain why *mxv* transcript was not detected in the *xre228* mutant. Alternatively, an unidentified *E. coli* inhibitor may be expressed in the *xre228* mutant.

### Differences between WT-T and tan mutants: protein kinases and Xre-type DNA-binding proteins

**Figure 6:**
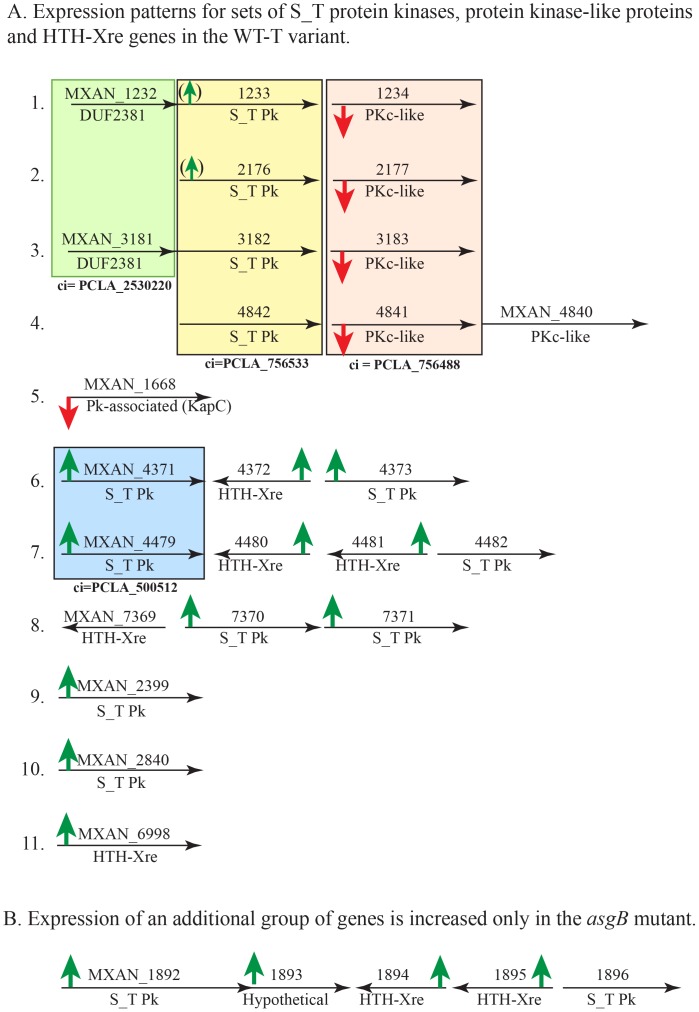
Expression of regulators and signal transduction proteins show phase-specific behavior. (A1-40 Four genes encoding nearly identical protein kinase-like proteins are down-regulated in WT-T variants. In each case, the PKc-like gene is downstream of a gene annotated as a serine-threonine protein kinase (S_T Pk). The PKc-like genes that share significant similarity with serine-threonine protein kinases. (A5) A putative *kapC* gene (KapC =  protein kinase associated protein) is down-regulated in WT-T. (A6–10) Expression of multiple S_T Pk-encoding genes and HTH-Xre-encoding genes (helix-turn-helix xenobiotic response element family of DNA-binding proteins) is increased in WT-T relative to WT-Y strains. Four colored boxes indicate genes whose products share sufficient identity to be considered a protein cluster (ci =  cluster index; NCBI protein cluster database PCLA designation). (B) The *asgB* mutant behaves similar to the WT-T with regard to genes in A, but differs from WT-T in that it also shows significant increases in four additional genes predicted to encode a S_T Pk, a hypothetical and two HTH-Xre proteins.

While the expression profiles of the *asgB*, *xre228* and *dkxG* mutants resemble that of the WT-T variant with regard to genes for iron acquisition, pigment production and antibiotic, other changes in gene expression reveal patterns that highlight differences between these strains. The genome of *M. xanthus* DK1622 contains over 50 genes annotated as serine-threonine protein kinases (S_TPk) or ST_kinase-like proteins (PKc-like) and as many as 24 genes encoding HTH-Xre DNA binding proteins. When we compared the transcriptomes of tan strains, a pattern emerged for a significant subset of the kinase and HTH-Xre-encoding genes. The data are presented in Table 3; the genomic context for these genes is shown in Fig 6.

**Table 3:** Clusters of ST_ kinases, PKc-like proteins and HTH-Xre players show altered expression during phase variation.

A. Genes differentially regulated in two or more tan strains					
		Fold change			
MXAN ID	Description	WT-T	*asgB*	*dkxG*	*xre228*
1233	serine-threonine kinase	+2	+3	−3	+1
1234	protein kinase-like (PKc-like)	−41	−55	−1	−12
1668	serine-threonine kinase associated protein KapC	−8	−16	+2	+2
2177	protein kinase-like (PKc-like)	−35	−62	+1	−24
2399	serine-threonine kinase	+11	+4	−1	+5
2840	serine-threonine kinase	+57	+32	−1	+1
3183	protein kinase like (PKc-like)	−37	−60	+1	−20
4371	serine-threonine kinase	+132	+293	−3	−15
4372	HTH-Xre DNA-binding protein	+96	+337	−1	−3
4373	serine-threonine kinase	+9	+9	1	−1
4479	serine-threonine kinase like	+43	+42	−5	−6
4480	HTH- Xre DNA-binding protein	+26	+51	−1	−1
4481	HTH- Xre DNA-binding protein	+31	+50	+1	−2
4841	protein kinase like (PKc-like)	−46	−72	+1	−17
6998	HTH- Xre DNA-binding protein	+109	+125	+2	+1
7370	serine-threonine kinase	+187	+293	+2	+2
7371	serine-threonine kinase	+99	+116	+10	+20

B. Genes up-regulated significantly only in the *asgB* mutant					
1892	protein kinase-like (PKc-like)	−1	+42	−2	−4
1893	hypothetical	+1	+354	+2	+3
1894	HTH-Xre DNA binding protein	+1	+46	+1	+1
1895	HTH-Xre DNA binding protein	+1	+148	+2	+2

Legend: A description of the microarray is provided in Table 1 legend. Column headings: ID = MXAN # and description refers to annotation; both are derived from the *M. xanthus* genome [43]; fold change represents the increase or decrease in expression and is derived from coefficient value. +  =  increased expression; -  =  decreased expression.

Expression of seven S_TPk, five *xre* genes, four PKc-like, and one *kap* (kinase associated protein) gene showed significant increases or decreases in the WT-T strain and some tan mutants. Expression of four protein kinase C-like proteins (PKc-like), MXAN_1234, 2177, 3183 and 4841, was decreased 41-, 35-, 37- and 46-fold, respectively in the WT-T variant compared with the WT-Y. Each of these genes encodes a member of the protein kinase-like superfamily (cl09925) and contains the catalytic domain of serine-threonine kinases. These four PKc-like proteins are in the PLCA_756488 identity cluster (Fig 6 pink box); they share >80% identity over the entire length (596–597 residues). Expression of all four PKc-like genes was decreased in the *asgB* and *xre228* mutants, but not in the tan *dkxG* mutant.

It is significant to note that the genomic context of these genes is very similar, which suggest that gene duplication occurred at some point. Each PKc-like gene is immediately downstream of a gene encoding a serine-threonine protein kinase whose expression was virtually unchanged in the tan strains. The four S-TPk proteins share significant identity (protein cluster 756533; Fig 6 yellow box). The sets of genes diagramed in Fig 6 (1–4), follow the pattern serine-threonine kinase (S_T Pk) and protein kinase-like (Pkc), respectively. In two cases, there is an additional, related gene upstream (Fig 6, green box). Expression of *MXAN_1668*, encoding a serine-threonine kinase associate protein (KapC; Fig 6 line 5), was decreased in WT-T and the *asgB* mutant, but was increased slightly in the *dkxG* and *xre228* mutants.

In contrast, expression was increased significantly in WT-T and the *asgB* mutant for seven serine-threonine protein kinase (S_T Pk)-encoding genes and five HTH-Xre encoding genes (Fig 6, 6–11). In some cases, the S_T Pk genes are adjacent to the HTH-Xre genes. As a rule, expression levels were higher in the *asgB* mutant than in the WT-T strain (Table 3). Except for *MXAN_7371*, expression of the S_T Pk and HTH-Xre genes in *dkxG* and *xre228* strains was unchanged (value  = 1), increased slightly (MXAN_6998) or even decreased (*MXAN_4371* and *4479*) which highlights some major distinctions between the tan strains.

Expression of four additional genes, related to the group described above, showed an increase unique to the *asgB* mutant. As shown in Fig 6B and Table 3B, expression of *MXAN_1892* (S_T Pk), *1893* (hypothetical), *1894* (HTH-Xre) and *1895* (HTH-Xre) was up-regulated 40 to 350-fold in the *asgB* mutant whereas the other three tan strains showed no change or a slight decrease in expression.

The expression profile of genes other than those in Tables 1, 2, and 3 is listed in Table 4. These genes fell into four groups: (A) those affected in WT-T *asgB* and *xre228*, (B) those affected in WT-T and *asgB*, (C) those affected in *asgB*, *dkxG*, and/or *xre228*, but not in WT-T, (D) those affected in *asgB* only and (E) those showing the opposite response in one or more tan strain. Group A includes several hypotheticals and acyl carrier protein. Group B (WT-T and *asgB*) includes hypotheticals, bacterial leucyl aminopeptidase and a cytochrome P450 family protein. Most of the genes in Group C, including a lipoprotein and phosphatase, were up-regulated significantly in *asgB* and to a lesser extent, in *dkxG*.

**Table 4:** Outliers: a subset of genes show differential expression profiles that may help distinguish pathways in tan strains.

MXAN ID	Description	WT-T	*asgB*	*dkx*	*xre228*
**Group A: changes in WT-T, ** ***asgB*** ** and ** ***xre228***					
3949	acyl carrier protein	−235	−172	−2	−112
1697	conserved hypothetical protein	−51	−130	−3	−65
3434	hypothetical protein	−23	−110	−2	−40
5266	hypothetical protein	−23	−64	−3	−27
**Group B: changes in WT-T and ** ***asgB***					
0519	conserved hypothetical protein	+10	+19	+1	+1
4985	hypothetical protein	+5	+55	+1	+1
5166	bacterial leucyl aminopeptidase	−23	−67	−1	−1
6991	hypothetical protein	−12	−49	−1	−2
7298	cytochrome P450 family protein	−8	−50	−1	−2
**Group C: changes in ** ***asgB, dkx*** **, and/or ** ***xre228*** ** but not in WT-T**					
0502	transcriptional regulator, AsnC family	+1	+1	+7	+12
1238	conserved hypothetical protein	+1	+97	+6	+1
1485	putative cytochrome c	+1	+70	+7	+1
1486	HNH endonuclease domain protein	+1	+114	+8	+1
1499	ThiF domain protein	+1	+21	+4	+1
1508	hypothetical protein	+1	+57	+9	+1
1509	Ser-Thr protein phosphatase family protein	+1	+92	+6	+1
3166	hypothetical protein	+1	+73	+5	+2
5986	putative lipoprotein	+1	+77	+21	+11
6350	hypothetical protein	+1	+35	+4	+1
**Group D: changes in ** ***asgB*** ** only**					
0383	hypothetical protein; C-terminal catalytic domain BreC (break-rejoining enzymes)	+1	+124	+3	+1
0384	quaternary ammonium compound-resistance protein SugE (*sugE*)	+2	+93	+3	+3
0455	conserved domain protein	+1	+35	+1	+1
1894	DNA-binding protein	+1	+46	+1	+1
2912	hypothetical protein	+2	+48	+2	+1
5350	DNA repair protein RecN (recN)	+2	+23	+1	+1
6264	conserved hypothetical protein	+2	+27	+1	+1
7217	hypothetical protein (BIg domain)	+5	+2383	+2	+5
7219	hypothetical protein (BIg domain)	+2	+1146	+7	+5
7222	kelch domain protein	+2	+12	+2	+1
**Group E: Opposite response in different tan strains**					
0046	hypothetical protein	+10	+11	−3	+1
0047	hypothetical protein	+9	+15	−3	+1
0048	hypothetical protein	+17	+33	−2	+1
0049	hypothetical protein	+11	+22	−1	+1
0050	hypothetical protein	+11	+12	−1	+2
0201	hydrolase, alpha-beta fold family	+71	+50	−2	+10
0503	DnaJ domain protein, authentic frameshift	−7	−133	+2	+8
0504	hypothetical protein	−12	−171	+2	+7
0587	trypsin domain protein	−8	−144	−1	+1
0947	RNA polymerase sigma-70 factor, ECF subfamily	+1	+11	−2	−3
1164	GAF domain protein	−10	−176	+1	−3
1650	peptidase, S1A (chymotrypsin) subfamily	−9	−99	+2	−2
1725	hypothetical protein	−11	−76	+0	+2
1893	hypothetical protein	+1	+356	−1	−3
2297	conserved hypothetical protein	+3	+142	−1	−1
3676	hypothetical protein	−11	−63	+1	−2
3985	hypothetical protein	+4	+35	+5	−1
4305	amino acid adenyltransferase	−52	−57	+1	−69
4368	hypothetical protein	+3	+24	+7	−3
4837	endoglucanase (*celA*)	−11	−105	−1	+1
5970	peptidase, S8 (subtilisin) family	−7	−111	+1	−2
7375	conserved hypothetical protein	+2	+29	−1	−1

Table legend: Column headings: fold increase represents the change in expression and is derived from coefficient value; ID = MXAN # and description refers to annotation; both are derived from the *M. xanthus* genome data at NCBI [43].

Group D (increased mainly in the *asgB* mutant) contained genes encoding proteins that contain Big (bacterial immunoglobulin-like; pfam13205) domains; some Big proteins have been shown to contain novel Ca^2+^ binding modules [25]. As shown in Table 4, expression of *big* genes *MXAN_7217* and *7219* was increased over 1000-fold in *asgB* while almost unchanged in the other tan strains. There were eight more genes that showed a significant (>10 fold) increase relative to the other three tan strains. These include *MXAN_0383* (hypothetical, DNA-BreC (breaking rejoining catalytic domain)) and its neighbor *MXAN _0384* (quaternary ammonium compound-resistance protein SugE). Finally, Group E genes were generally increased or decreased in WT-T and *asgB* but showed the opposite response in *dkxG* and/or *xre228*. For example, *MXAN*_ 0503 and 0504 were decreased in WT-T and *asgB* but increased in *dkxG* and *xre228*.

## Discussion

Iron is an essential element for microbial growth. Among other things, it is necessary for enzymatic function of ribonucleotide reductase, iron-sulfur proteins, succinate dehydrogenase, and heme-containing enzymes such as cytochromes and peroxidases. The redox versatility of this element accounts for its biological significance but also contributes to problems with acquisition of iron. This is particularly true for microbes such as *M. xanthus* that thrive in aerobic environments because Fe is very insoluble at neutral pH and in oxic environments (available Fe ≈10^−17^ M). Most microbes produce extracellular ligands such as siderophores to sequester needed iron from the environment and have dedicated transport systems to bring Fe (III) siderophore complexes into the cell.

The results of this study demonstrate that iron availability plays a role in phase variation of *Myxococcus xanthus*. First, comparison of otherwise isogenic yellow and tan variants revealed that tan variants up-regulate a core set of genes encoding proteins critical for acquisition of iron, including enzymes for synthesis of the siderophore myxochelin (*mxc*) and import of Fe•myxochelin and hemin. Biochemical assays confirmed the increase in siderophore in tan variants. Second, addition of iron dramatically inhibited expression of *mxc* genes and slowed the yellow-to-tan switch significantly, effectively “prolonging” cells in the yellow phase. When the concentration of available iron was reduced, the number of tan variants arising from a wild-type yellow culture increased.

**Figure 7:**
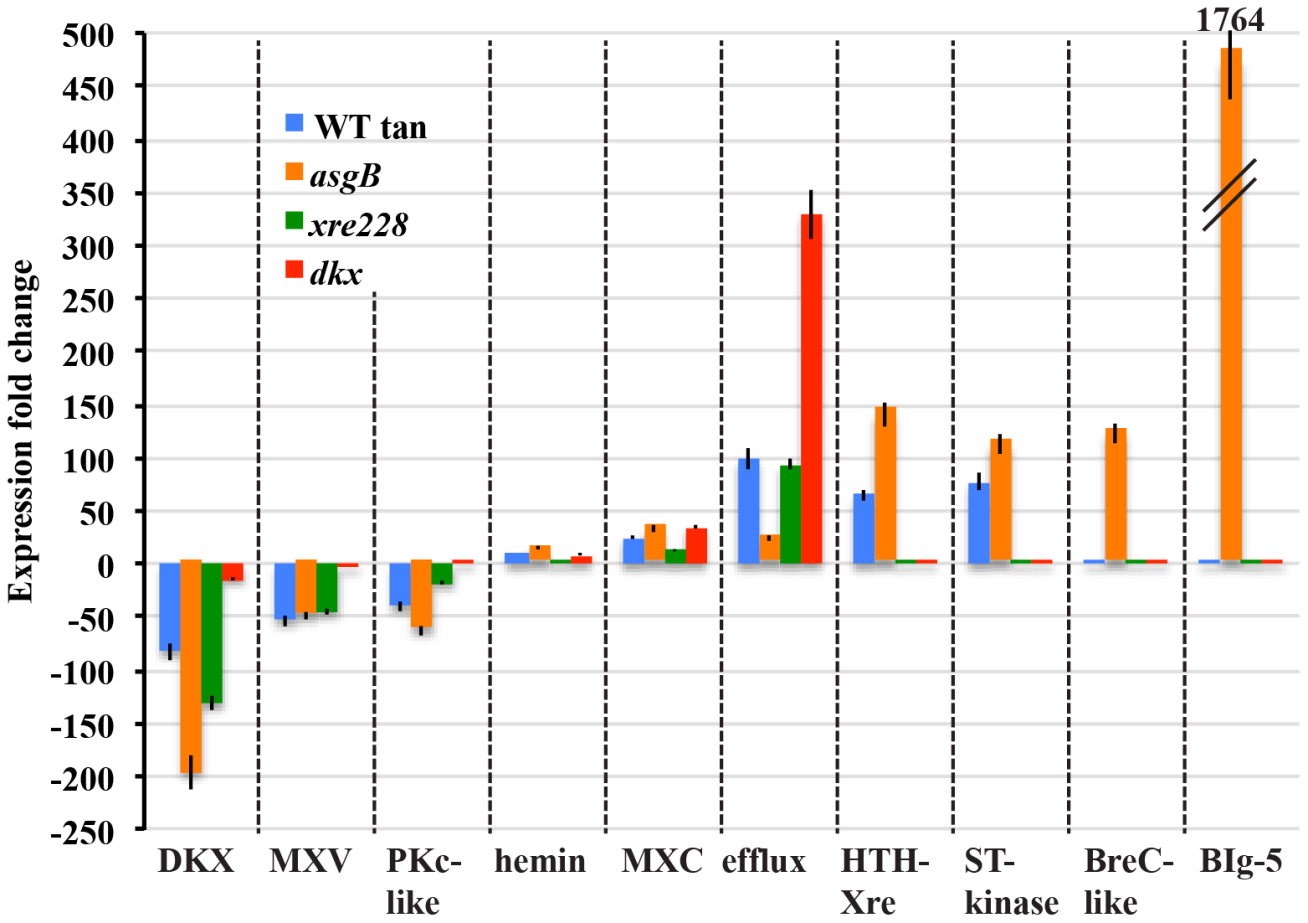
Potential biomarkers of phase variation. A side-by side comparison illustrates the magnitude of changes between the tan variant and the mutants and highlights some of the outliers. Eight of the ten markers (genes or operons) show significant changes in the WT-T, while the remaining two genes represent changes that are unique to the *asgB* mutant. The fold change for gene operons represents the average of all genes in the pathway. DKX represents the average of the 14 DKxanthene genes (*MXAN_4289-4305*), MXV represents the average of 21 myxovirescin genes (*MXAN_3930-3950*), pKc represents the average of four protein kinase-like genes (*MXAN_1234, 2177, 3183, 4841*), hemin represents the average of nine myxochelin genes (*MXAN_3639-3647*), efflux represents the average of four macrolide efflux genes (*MXAN_4198-4201*), HTH-Xre represents the average of four xenobiotic response element genes (*MXAN_4372, 4480, 4481, 6998*), and ST_kinase represents the average of seven protein kinases (*MXAN_2399 2840, 4371, 4373, 4479, 7370, 7371*). BreC is *MXAN_0383* and BIg-5 is the average of *MXAN_7217* and *7219*, which at fold increase of 1764, was off the scale of this graph.

**Figure 8:**
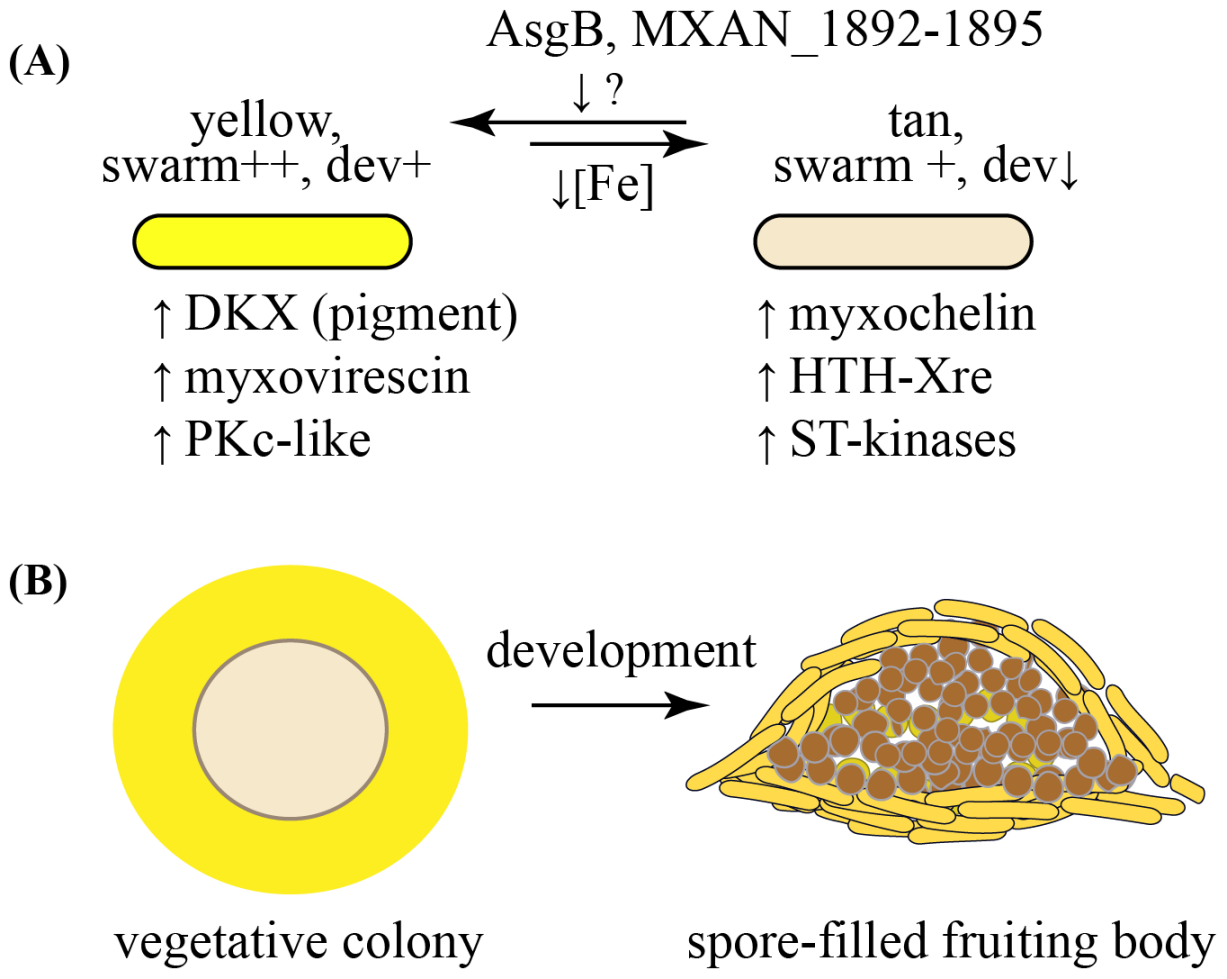
Phase variation yields two cell types. (A) The data show that the concentration of iron influences the yellow to tan phase switch; in this model, we propose that AsgB or AsgB-dependent products play a key role in the reverse direction switch. The yellow variants produce myxovirescin, the antibiotic needed for predation [23]. Tan variants are mediocre swarmers but are more adept at sequestering iron. (B) The division of labor between yellow and tan variants may be critical for survival of *M xanthus* in nature. The robust yellow swarmers are enriched at the periphery of a WT colony whereas slow swarming tan variants would be more enriched at the colony center. The ramifications for development are clear: tan cells, which are predisposed to become spores are well positioned to end up in the interior of the fruiting body and are surrounded by cells that produce DKX, the pigment needed to for production of viable spores [13].

Iron acquisition genes were not the only genes responsive to phase variation. As shown in Fig 7, expression of PV markers, including DKX, antibiotic myxovirescin (Mxv), and kinase-like proteins (PKc-like) decreased while expression of potential regulators, serine threonine kinases and HTH-Xre proteins, increased in WT-T (blue bars). Changes in Mxv and DKX may reflect a metabolic trade-off as cells cease to invest in secondary metabolites when there is shortage of iron. Surprisingly, although swarming is also diminished in tan variants compared with yellow variants, we did not find differences in expression of known gliding genes. General characteristics of yellow and tan variants are summarized in Fig 8A.

To begin to understand the context for these PV markers, three strains that are tan due to mutation were analyzed alongside the WT-T. The *dkx* mutant (Fig 7 red bars), which is tan due to disruption of the pigment biosynthetic pathway, resembled the WT-T expression profile for *mxc*, hemin, and macrolide efflux genes. Hence, loss of pigment or disruption of the pigment biosynthetic pathway alone is sufficient to activate the iron acquisition scheme in *M. xanthus*. The DKX pathway does not autoregulate because disruption of the *dkxG* gene did not affect expression of genes in other *dkx* operons. Loss of DKX did not affect production of Mxv or PKc-like proteins, however.

The overall profile of the *xre228* mutant (Fig 7 green bars) positions the function of Xre228 ahead of *dkx* and suggests that Xre228 regulates *dkx*, *mxv*, and genes encoding the four PKc-like proteins. Several key differences between *xre228* and WT-T helped pinpoint the position of Xre228 in the PV pathway. Specifically, clusters of genes including *MXAN_4371-4373*, *4479-4481*, and *7370* were unchanged in the *xre228* mutant, which suggests that the ST-kinases and HTH-Xre act before Xre228 in the PV pathway or are independent of Xre228.

The pattern of the *asgB* mutant (Fig 7 orange bars) paralleled the WT-T strain for the eight PV biomarkers but showed substantial increases in genes such as *breC* (putative break-rejoining enzyme) and *big* (Fig 7) and potential regulators (Fig 6B). AsgB is a DNA-binding protein involved in cell-density dependent signaling [26]. It contains a small (≈30 amino acids), C-terminal HTH-Xre superfamily domain, similar to Xre228 and conserved in region 4 of the sigma 70 proteins (pfam04545) [15]. Although *asgB* was predicted to be essential [15] we were able engineer stable disruptions of the gene. Additionally, while the *asgB* mutant has historically been described as a tan mutant, our Kan^R^ *asgB* mutant was initially yellow, but it converted to tan over the period of a few weeks after which time it was a stable tan mutant. This result, combined with similarities in the transcriptome and phenotype data between the WT-T and the *asgB* mutant lead us to speculate that *asgB* mutant is blocked in the ability to switch from tan to yellow, as shown in Fig 8A. If this model is correct, AsgB or the genes it controls, including *MXAN_1892-1895* (S_TPk and HTH-Xre proteins) may govern one of the main PV switch pathways. The severity of the *asgB* developmental phenotype may relate to the fact that it is locked in the tan phase. The BreC and BIg-domain proteins whose expression AsgB regulates may play direct roles in PV or may affect developmental processes that are known to be part of a signaling pathway regulated by AsgB [27].

Bacterial serine-threonine protein kinases were first discovered in *M. xanthus* [28] and have been shown to play critical roles in the complex life cycle of this organism [29], [30]. Two types of kinases, PKc-like and S_TPk, exhibited divergent regulation during PV. Genes for four nearly identical PKc-like proteins were down-regulated in WT-T, *asgB* and *xre228*. Gene expression was repressed to a similar extent for each gene, which suggests that their regulation may be coordinated and that they may encode redundant functions. The similarity between these proteins and the proximity of each to a S_TPk suggests that these clusters are the result of gene duplication. Such regions of the chromosome may be targets for gene conversion, a known mechanism for regulating phase variation. In contrast with the PKc-like genes, expression of S_TPk genes was increased in WT-T and *asgB*. This group of S_TPk may function in pathways involving motility or development, which are impacted in WT-T and *asgB*.

The ability of *M. xanthus* to generate two distinct cell types depending on the availability of iron makes sense given the social nature and habitats of this organism. *M. xanthus* has been compared to higher order social organisms, including bees and ants, that live in large groups and cooperate through a division of labor to accomplish complex tasks. As *M. xanthus* colonies normally contain a mixture of yellow (robust swarmers) and tan (mediocre swarmers) cell types, phase variation takes on a mutualistic quality. During growth, yellow variants accumulate at the colony edge and surround the slower swarming tan variants (Fig 8B). Elevated levels of DKX and Mxv, which are needed for sporulation and predation, respectively, likely benefit tan variants. In return, elevated levels of Fe•siderophore produced by tan variants may benefit yellow variants. When nutrient depletion induces the development cycle, tan variants enriched near the colony center and bathed in chemicals, such as DKX, from yellow variants are well positioned to differentiate into spores. Maintenance of both yellow and tan cell types with minimal ‘cheating’ may be safeguarded by the fact that the potentially iron-rich tan cells need DKX for efficient spore production. As only yellow cells produce the DKX pigment, their survival is guaranteed.

## Materials and Methods

### Bacterial strains and growth conditions

A list of strains, plasmids, and oligonucleotides is provided in Table S2. The *M. xanthus* strains included wild-type strain DK1622 [31]. DK1622 shows characteristic normal gliding motility, swarming, fruiting body morphogenesis, sporulation, and phase variation and was used as the source for yellow and tan phase cells. The *asgB* gene was disrupted using the Mip (myxo integrative plasmid) technique.

*M. xanthus* was grown routinely at 32°C at >200 rpm in CTPM liquid medium (1% Casitone, 10 mM Tris, 1 mM potassium phosphate, 5 mM MgSO_4_, final pH 7.6); according to the manufacturer, a 1% solution of Casitone is 15–45 µM Fe. TPM buffer is identical except that it lacks Casitone. CTPM was supplemented with kanamycin (Kan; 40 µg/ml) when applicable. Dipyridyl (2, 2-dipyridyl) is a bidentate chelating ligand that was added to CTPM medium to deplete iron. Where applicable, CTPM medium was supplemented with ferric citrate, ferric chloride, copper chloride, or dipyridyl. LB was used for growth of *E. coli*.

### Analysis of wild-type phase variants and mutants

To determine viable cell numbers, aliquots were removed from broth cultures at regular intervals and plated on rich medium. Colony forming units (CFU) were determined by removing aliquots of cells and diluting serially 1∶10 in CTPM broth. Viability was checked by plating 4 µl spot aliquots of each dilution or by spread plating 50 µl aliquots of the 10^−4^ to 10^−5^ samples. After 4 days growth at 32°C, plates were scored for growth on spot plates and total number of yellow and tan colonies on spread plates. The swarming rate of each mutant was compared with the WT strain on CTPM medium with 0.3% and 1.5% high grade agar as described elsewhere [32]. Cell aggregation, Congo red and trypan blue dye binding were assayed using published protocols [9].

Siderophore: The amount of siderophore in supernate and cell pellet was determined by the liquid CAS assay [22]. Cells were grown to a density of 5×10^8^ cells ml^−1^ (Klett = 100) in CTPM (+ supplements where appropriate). A 1 ml aliquot was removed and centrifuged in a microfuge tube for 2 min. The supernate was transferred to a fresh tube. The cell pellet was suspended in 1 ml of 1% NP-40 and resuspended by pipetting up and down; samples were incubated for 5 min to completely lyse the cells. Aliquots (0.4 ml each) of supernate and lysed cell suspension were transferred to glass tubes and 0.4 ml of CAS-Fe-HDTMA piperazine assay solution (below) was added to each tube and vortexed immediately. The CAS controls contained 0.4 ml CTPM broth (+ supplements where appropriate) or 1% NP-40. Tubes were incubated at room temperature for 30 min. After reaching equilibrium the absorbance was measured at 630 nm and compared against the cell free controls.

CAS-Fe-HDTMA piperazine solution: the liquid CAS solution was prepared as follows: 7.5 ml of 2 mM CAS was mixed with 1.5 ml of 1 mM FeCl_3_•6 H_2_O, and 50 ml HDTMA was added with stirring. The CAS-Fe-HDTMA was brought to 100 ml total volume by addition of piperazine buffer (≈36–40 ml, depending on the about of HCl used to bring pH to 5.6) and H_2_O to balance. Piperazine buffer was prepared by dissolving 9.7 gm piperazine hexahydrate (or 4.3 gm piperazine) in 30 ml H_2_O and adjusting the pH to 5.6 with conc HCl; the final volume was 37–40 ml.

Chrome azurol S (CAS) was added to CTPM medium to quantify the production of siderophore on agar medium. Sterile CAS•Fe• hexadecyltrimethylammonium bromide (HDTMA) [33] was added to CTPM agar. Aliquots of *M. xanthus* strains were spotted on CAS-Fe-HDMA medium and incubated for >18 hrs at 32°C and the zone of color was measured (growth did not occur as cells are sensitive to the HDTMA detergent).

DKxanthene: The amount of DKX was estimated by spectral analysis of log-phase cells (density noted for each experiment) harvested by centrifugation and suspended in 1 ml of TPM. DKX was measured at 410 nm (λ_max_ [13]) using a Spectronic 21D spectrophotometer (Milton Roy).

Changes in siderophore and Abs _410 nm_ (λ_max_ for DKX) in response to different concentration/types of metals were measured from cells grown in CTPM supplemented with different components. First, CTPM broth-grown WT-Y and WT-T cells (density 4×10^8^ cells ml^−1^) were diluted 1∶50 in CTPM and immediately divided into four aliquots. Aliquot #1 received H_2_O, #2 received FeCl_3_ (100 µM fc), #3 received dipyridyl (25 µM fc) and #4 received CuCl_2_ (100 µM fc). Each of the four aliquots was split into two culture flasks and incubated at 32°C with shaking until reaching a density of 6×10^8^ cells ml^−1^. Aliquots were removed and assayed for siderophore and absorbance at 410 nm as described above. Siderophore and Abs_410_ values for the test samples were compared against the original inoculum (density 4×10^8^ cells ml^−1^) used for each series. Add-back experiments were performed with a basal medium of CTPM+25 µM dipyridyl to which H_2_O, CuCl_2_, FeCl_3_ or ZnCl_2_ was added at amounts listed in the text.

Mxv: The production of myxovirescin was determined using the myxovirescin assay described by [24] with the following modifications. Cultures of *M. xanthus* were harvested by centrifugation and suspended in TPM buffer to 5×10^8^ cells ml^−1^ (Klett 100). 10 µl aliquots were spotted on TPM agar medium with 0.5% casitone and incubated at 32°C. After 18 hr, plates were overlaid with 3 ml of 0.5% CTPM soft agar containing 100 µl of an overnight culture of *E. coli* DH5α. The zone of inhibition (ZOI) was measured after 24 hr incubation at 32°C and is reported as the diameter of the clearing zone.

### Microarray analysis

A custom Roche NimbleGen 12-plex microarray was designed for *M. xanthus* based on a consensus sequence from mapping paired end 91 bp Illumina sequence data from the National Genome Research Center for two tan and two yellow clones to the reference genome (dk_1622). Use of this custom array made it possible to generate more refined transcriptome data than previously [9]. The microarray contained 137,799 60-mer probes tiled across the genome, with an average spacing of 65 bp, and representing a possible 6,935 expressed transcripts (probe sets). Probe sets were defined as all probes that mapped to a single gene. Double-stranded cDNAs were synthesized using Invitrogen's SuperScript cDNA Synthesis Kit with the standard protocol using oligo dT primers (Invitrogen, Carlsbad, CA). RNA was harvested from yellow and tan derivatives of the WT (DK1622) and mutants as described previously [9]. cDNAs were fluorescently labeled with Cy3 using the standard NimbleGen Gene Expression Analysis protocol. All samples were hybridized to a single 12-plex chip to reduce technical noise. An 18-hour hybridization was conducted at 42 degrees in a NimbleGen Hybridization System 4 chamber (NimbleGen, Madison, WI). The chip was washed and then scanned on a Roche MS200 scanner. Finally, probe signal intensities were extracted using Roche Nimblegen's NimbleScan Software v2.6. Samples with adjusted p values of <0.05 from the independent array runs (triplicate samples of three biological replicates) were considered statistically significant. All analyses were done in the R programming language [34], raw intensity data were read in and checked for quality using the Limma package [35]. The data were then background corrected using background subtraction and the arrays were normalized with quantile normalization [36]. A simple linear model was fit to the data and variances were corrected using an empirical Bayes approach [37] from the bioconductor limma package. Results were corrected for multiple testing using the false discovery rate procedure of Benjamini and Hochberg (BH) and considered significant if their BH adjusted p-values were <0.05 [38].

### Real-time PCR

WT–Y, WT-T variants and mutants were grown to a density of 5×10^8^ cells ml^−1^ in CTPM medium at 32°C. A 1 ml aliquot was harvested by centrifugation at 13,700x*g* and the cell pellet was suspended in 0.5 ml PBS buffer. Total mRNA was extracted using RNAprotect Bacterial reagent (with DNase) and RNeasy minikit according to manufacturers instruction (Qiagen, Valencia CA). 500 ng total RNA was used to produce cDNA using a Hexonucleotide Mix (Roche, Indianapolis, IN) as primer for the reaction using the Superscriptase II kit (Invitrogen). The resulting cDNA was diluted 1∶25 or 1∶1250 for probing target genes and 16s rRNA templates respectively. 5 µl of diluted cDNA sample was added to 20 µl of a PCR mixture prepared from Power SYBR Green PCR Master Mix (Applied Biosystems, Carlsbad, CA), which contained each primer at a concentration of 160 nM. Primers, listed in Table S1, were chosen using Primer Express 3.0 software (Applied Biosystems) and were designed to amplify a region of about 150–200 bp within each transcript.

## Supporting Information

Fig. S1 Cell cohesion (*aka* agglutination) is a phenotype that can be used to distinguish yellow and tan variants. The cohesion assay was performed as described in Methods. Tan strains (WT-T, *asgB*, *xre228*, and *dkxG*) were compared with the WT-Y variant; data are presented for three independent assays.
(TIF)

Fig. S2 The WT-T (right) strain produces a larger siderophore halo than the WT-Y (left) strain. Cultures were grown to 5×10^8^ cells ml^−1^ (Klett 100) and concentrated 10-fold in TPM buffer. 5 µl aliquots were spotted on CTPM blue agar (containing CAS-Fe) and incubated at 32°C for 4 hr.
(TIF)

Table S1 DNA microarray data supplement to Tables 1 and 2. Additional data are listed in Tables 3 and 4. Supporting array table legend: Genes showing significant expression changes in at least one of the *M. xanthus* tan strains (12 datasets: four biological replicates with triplicate technical replicates) in DNA tiling array studies relative to the WT-Y variant. Column headings: fold increase represents the change in expression and is derived from coefficient value; ID = MXAN # and description refers to annotation; both are derived from the *M. xanthus* genome data at NCBI http://www.ncbi.nlm.nih.gov/genome/1120.
(DOCX)

Table S2 Strains and molecular reagents.
(DOCX)
