# Supplementary figures and images for: Phase Variation in Myxococcus xanthus Yields Cells Specialized for Iron Sequestration
Source: PLoS One. 2014 Apr 14;9(4):e95189. doi: 10.1371/journal.pone.0095189 (PMC3986340; doi:10.1371/journal.pone.0095189)

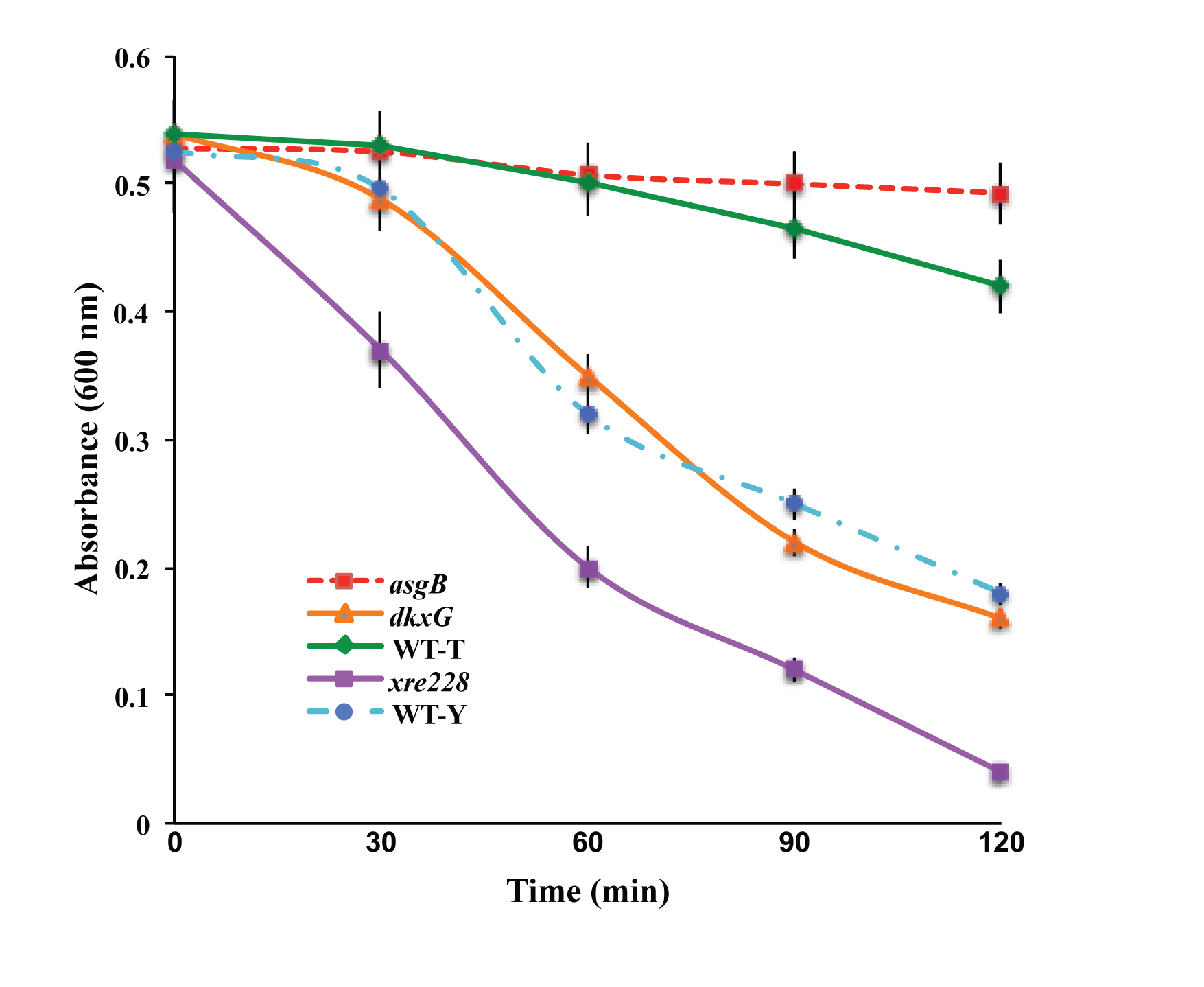

Supplement: Fig. S1 — Cell cohesion (aka agglutination) is a phenotype that can be used to distinguish yellow and tan variants. The cohesion assay was performed as described in Methods. Tan strains (WT-T, asgB, xre228, and dkxG) were compared with the WT-Y variant; data are presented for three independent assays. (TIF) [file pone.0095189.s001.tif]

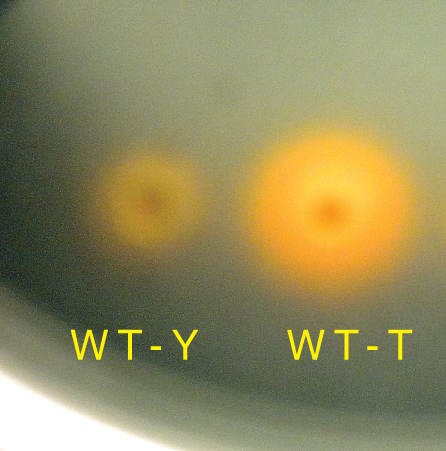

Supplement: Fig. S2 — The WT-T (right) strain produces a larger siderophore halo than the WT-Y (left) strain. Cultures were grown to 5×10^8^ cells ml−1 (Klett 100) and concentrated 10-fold in TPM buffer. 5 µl aliquots were spotted on CTPM blue agar (containing CAS-Fe) and incubated at 32°C for 4 hr. (TIF) [file pone.0095189.s002.tif]
