# Supplementary material for: Phase Variation in Myxococcus xanthus Yields Cells Specialized for Iron Sequestration
Source: PLoS One. 2014 Apr 14;9(4):e95189. doi: 10.1371/journal.pone.0095189 (PMC3986340; doi:10.1371/journal.pone.0095189)
Supplement: Table S1 — DNA microarray data supplement to Tables 1 and 2. Additional data are listed in Tables 3 and 4. Supporting array table legend: Genes showing significant expression changes in at least one of the M. xanthus tan strains (12 datasets: four biological replicates with triplicate technical replicates) in DNA tiling array studies relative to the WT-Y variant. Column headings: fold increase represents the change in expression and is derived from coefficient value; ID = MXAN # and description refers to annotation; both are derived from the M. xanthus genome data at NCBI http://www.ncbi.nlm.nih.gov/genome/1120. (DOCX) [file pone.0095189.s003.docx]

**Table S1: DNA microarray data supplement to Tables 1 and 2. Additional data are listed in Tables 3 and 4.**

| **A. Genes whose expression is increased relative to WT-Y** | | | | | **Fold change in** | | | | | |
| --- | --- | --- | --- | --- | --- | --- | --- | --- | --- | --- |
| **MXAN ID** | | | **Description** | | **WT-T** | ***asgB*** | ***dkx*** | ***xre228*** | | |
| **transporters, receptors, metal binding gene clusters** | | | | | | | | | | |
| MXAN_1316 | | | | putative TonB dependent receptor (BtuB, FepA, TomB-hemin, OM_channels superfamily, ligand-gated channel) OM receptor | +8 | +24 | +13 | +5 | | |
| MXAN_1317 | | | | putative lipoprotein | +12 | +19 | +11 | +6 | | |
| MXAN_1318 | | | | hemin transport protein HemS (*hemS*) (ChuX_HutX superfamily) | +13 | +17 | +10 | +6 | | |
| MXAN_1319 | | | | hemin ABC transporter, periplasmic hemin-binding protein | +10 | +11 | +9 | +5 | | |
| MXAN_1320 | | | | hemin ABC transporter, permease protein (*hemU*) | +8 | +8 | +6 | +4 | | |
| MXAN_1321 | | | | hemin ABC transporter, ATP-binding protein | +13 | +10 | +6 | +6 | | |
| MXAN_1688 | | | | TonB family protein | +6 | +20 | +16 | +5 | | |
| MXAN_1689 | | | | conserved hypothetical protein | +17 | +17 | +11 | +8 | | |
| MXAN_1690 | | | | putative esterase | +20 | +34 | +10 | +6 | | |
| MXAN_2251 | | | | glycine betaine-L-proline ABC transporter, periplasmic substrate-binding protein | +7 | +43 | +4 | +3 | | |
| MXAN_3915 | | | | TonB family protein | +1 | +24 | +1 | +1 | | |
| **myxochelin operon** | | | | | | | | | | |
| MXAN_3639 | | | | iron-chelator utilization protein (MxcB) | +8 | +34 | +20 | +8 | | |
| MXAN_3640 | | | | siderophore biosynthesis aminotransferase MxcL (AAT_I superfamily, HemL) | +23 | +15 | +18 | +13 | | |
| MXAN_3641 | | | | major facilitator family transporter (efflux pump MxcK) (MFS) | +44 | +32 | +24 | +12 | | |
| MXAN_3642 | | | | 3-deoxy-7-phosphoheptulonate synthase (AroAA5 *Stigmatella aurantiaca* a15) | +55 | +39 | +37 | +16 | | |
| MXAN_3643 | | | | non-ribosomal peptide synthase (MxcG) | +32 | +24 | +29 | +10 | | |
| MXAN_3644 | | | | isochorismatase (MxcF) | +36 | +46 | +48 | +17 | | |
| MXAN_3645 | | | | 2,3-dihydroxybenzoate-AMP ligase (MxcE) | +16 | +39 | +41 | +15 | | |
| MXAN_3646 | | | | isochorismate synthase (MxcD) | +10 | +31 | +46 | +13 | | |
| MXAN_3647 | | | | 2,3-dihydro-2,3-dihydroxybenzoate dehydrogenase (MxcC) | +5 | +39 | +48 | +9 | | |
| **macrolide efflux** | | | | | | | | | | |
| MXAN_4198 | | | | putative outer membrane macrolide efflux protein | +75 | +26 | +374 | +86 | | |
| MXAN_4199 | | | | putative macrolide efflux ABC transporter, ATP-binding protein | +115 | +35 | +412 | +137 | | |
| MXAN_4200 | | | | putative macrolide-specific efflux protein MacA | +119 | +22 | +313 | +93 | | |
| MXAN_4201 | | | | putative macrolide efflux ABC transporter, permease protein | +88 | +15 | +216 | +60 | | |
| **iron transporters** | | | | | | | | | | |
| MXAN_6000 | | | | Fe-ABC periplasmic iron compound-binding protein transporter | +10 | +17 | +10 | +7 | | |
| MXAN_6911 | | | | FepA-like TonB-dependent receptor | +61 | +278 | +39 | +6 | | |
| **hypothetical, conserved hypothetical** | | | | | | | | | |  |
| MXAN_2248 | | | | conserved hypothetical protein | +8 | +40 | +1 | +1 | | |
| MXAN_5022 | | | | conserved hypothetical protein | +6 | +9 | +4 | +7 | | |
| MXAN_5093 | | | | conserved hypothetical protein | +11 | -1 | +3 | +9 | | |
| MXAN_6640 | | | | hypothetical protein | +12 | +44 | +14 | +3 | | |
| MXAN_6641 | | | | hypothetical protein | +25 | +103 | +41 | +7 | | |
| **lipoproteins** | | | | | | | | | |  |
| MXAN_6263 | | | | conserved hypothetical protein | +12 | +87 | +23 | +5 | | |
| MXAN_6613 | | | | putative lipoprotein | -2 | -2 | +29 | +2 | | |
| **miscellaneous** | | | | | | | |  |  |  |
| MXAN_0401 | | | | hypothetical protein | +2 | +23 | +2 | +5 | | |
| MXAN_0506 | | | | monooxygenase, flavin-containing | +9 | +429 | +12 | +4 | | |
| MXAN_0934 | | | | protease DO family protein | +6 | +13 | +17 | +5 | | |
| MXAN_1200 | | | | site-specific recombinase, phage integrase family | +4 | +26 | +4 | +2 | | |
| MXAN_3702 | | | | transcriptional regulator, Fur family | +2 | +3 | +2 | +2 | | |
| MXAN_4389 | | | | catalase KatB (*katB*) | +30 | +30 | +17 | +14 | | |
| MXAN_6714 | | | | methionine aminopeptidase, type I (*map*) | +27 | +4 | +6 | +4 | | |
| MXAN_6805 | | | | ribosomal protein S4 (*rpsD*) | +10 | +37 | +24 | +6 | | |
| MXAN_6806 | | | | putative hydrolase | +4 | +73 | +16 | +3 | | |
| MXAN_6967 | | | | transcriptional regulator, Fur family | +4 | +3 | +3 | +2 | | |
| **B. Genes whose expression is typically decreased in tan strains relative to WT-Y** | | | | | | | | | | |
| **ID** | | | | **Description** | **WT-T** | ***asgB*** | ***dkx*** | ***228*** | | |
| **DKxanthene biosynthesis** | | | | | | |  |  | | |
| MXAN_4289 | | | | hypothetical protein | -136 | -227 | -2 | -147 | | |
| MXAN_4290 | | | | putative thioesterase | -316 | -276 | -1 | -254 | | |
| MXAN_4291 | | | | conserved hypothetical protein | -185 | -289 | -1 | -177 | | |
| MXAN_4292 | | | | polyketide synthase | -62 | -140 | -4 | -111 | | |
| MXAN_4293 | | | | hypothetical protein | -32 | -584 | -3 | -169 | | |
| MXAN_4296 | | | | non-ribosomal peptide synthetase | -56 | -414 | -4 | -295 | | |
| MXAN_4297 | | | | polyketide synthase | -47 | -302 | -2 | -190 | | |
| MXAN_4298 | | | | polyketide synthase type I | -45 | -69 | -34 | -64 | | |
| MXAN_4299 | | | | non-ribosomal peptide synthase-polyketide synthase | -63 | -121 | -41 | -100 | | |
| MXAN_4301 | | | | polyketide synthase type I | -48 | -77 | -37 | -75 | | |
| MXAN_4302 | | | | FAD-binding domain protein | -55 | -114 | -59 | -112 | | |
| MXAN_4303 | | | | type I phosphodiesterase-nucleotide pyrophosphatase family protein | -42 | -73 | -1 | -86 | | |
| MXAN_4304 | | | | acyl-CoA dehydrogenase | -19 | -21 | -1 | -28 | | |
| MXAN_4305 | | | | amino acid adenyltransferase | -35 | -41 | -1 | -45 | | |
| **myxovirescin (antibiotic TA) synthesis** | | | | | | |  |  | | |
| MXAN_3930 | | | | Signal peptidase II (*lspA*) | -6 | -6 | -2 | -5 | | |
| MXAN_3931 | | | | conserved domain protein | -74 | -103 | -4 | -36 | | |
| MXAN_3932 | | | | polyketide synthase | -15 | -18 | -3 | -15 | | |
| MXAN_3933 | | | | mixed type I polyketide synthase - peptide synthetase | -17 | -20 | -4 | -176 | | |
| MXAN_3934 | | | | PfaD family protein | -30 | -49 | -4 | -34 | | |
| MXAN_3935 | | | | non-ribosomal peptide synthase-polyketide synthase Ta1 | -12 | -14 | -4 | -13 | | |
| MXAN_3936 | | | | polyketide synthase | -7 | -8 | -3 | -8 | | |
| MXAN_3937 | | | | luciferase-like monooxygenase family protein | -16 | -20 | -4 | -19 | | |
| MXAN_3938 | | | | polyketide synthase | -6 | -7 | -2 | -7 | | |
| MXAN_3939 | | | | enoyl-CoA hydratase-isomerase family protein | -17 | -21 | -3 | -19 | | |
| MXAN_3940 | | | | enoyl-CoA hydratase-isomerase family protein | -5 | -6 | -2 | -5 | | |
| MXAN_3941 | | | | polyketide synthase | -12 | -12 | -3 | -18 | | |
| MXAN_3942 | | | | acyltranferase-malonyl CoA-acyl carrier protein transacylase (*fabD*) | -20 | -25 | -2 | -22 | | |
| MXAN_3943 | | | | cytochrome P450 family protein | -77 | -79 | -2 | -44 | | |
| MXAN_3944 | | | | signal peptidase II (*lspA*) | -13 | -12 | -1 | -6 | | |
| MXAN_3945 | | | | polyketide TA biosynthesis protein TaF (*taF*) | -35 | -39 | -3 | -34 | | |
| MXAN_3946 | | | | putative acyl carrier protein | -164 | -195 | -2 | -146 | | |
| MXAN_3947 | | | | conserved hypothetical protein | -28 | -27 | -2 | -27 | | |
| MXAN_3948 | | | | polyketide TA biosynthesis protein TaC (*taC*) | -111 | -112 | -3 | -96 | | |
| MXAN_3949 | | | | acyl carrier protein | -236 | -172 | -2 | -112 | | |
| MXAN_3950 | | | | NusG-like protein (*taA*) | -208 | -79 | -2 | -95 | | |
| **transporters, receptors** | | | | | | |  |  | | |
| MXAN_4746 | | | | TonB-dependent receptor | -19 | -18 | -2 | -1 | | |
| MXAN_4747 | | | | putative lipoprotein | -7 | -16 | -2 | -1 | | |
| MXAN_7147 | | | | efflux transporter, RND family, MFP subunit | -6 | -2 | -6 | -7 | | |
| **putative operon** | | | | | | |  |  | | |
| MXAN_6223 | | | | sensor histidine kinase | -3 | -3 | -3 | -4 | | |
| MXAN_6225 | | | | putative lipoprotein | -4 | -25 | -27 | -18 | | |
| MXAN_6226 | | | | hypothetical protein | -6 | -27 | -47 | -24 | | |
| MXAN_6227 | | | | putative lipoprotein | -3 | -3 | -3 | -4 | | |
| **hypothetical, conserved hypothetical genes** | | | | | | |  |  | | |
| MXAN_0138 | | | | conserved hypothetical protein | -5 | -8 | -7 | -4 | | |
| MXAN_1019 | | | | hypothetical protein | -5 | -8 | -7 | -4 | | |
| MXAN_1592 | | | | hypothetical protein | -15 | -12 | -7 | -2 | | |
| MXAN_1697 | | | | conserved hypothetical protein | -52 | -131 | -3 | -65 | | |
| MXAN_2412 | | | | hypothetical protein | -9 | -5 | -11 | -11 | | |
| MXAN_3433 | | | | conserved hypothetical protein | -19 | -35 | -2 | -24 | | |
| MXAN_4492 | | | | conserved hypothetical protein | -9 | -11 | -2 | -13 | | |
| MXAN_5434 | | | | hypothetical protein | -7 | -13 | -3 | -5 | | |
| MXAN_6272 | | | | conserved hypothetical protein | -3 | -2 | -6 | -3 | | |
| MXAN_7409 | | | | conserved hypothetical protein | -3 | -6 | -6 | -9 | | |
| **lipoproteins, putative lipoproteins** | | | | | | |  |  | | |
| MXAN_0538 | | | | putative lipoprotein | -20 | -31 | -22 | -7 | | |
| MXAN_6563 | | | | putative lipoprotein | -4 | -7 | -3 | -4 | | |
| **miscellaneous** | | | | | | |  |  | | |
| MXAN_0996 | | | | tryptophan halogenase | -7 | -9 | -3 | -8 | | |
| MXAN_2791 | | | | protease B (*prtB*) | -6 | -62 | -8 | -4 | | |
| MXAN_6205 | | | | microbial collagenase | -17 | -34 | -10 | -7 | | |

Supplemental array table legend: Genes showing significant expression changes in at least one of the *M. xanthus* tan strains (12 datasets: four biological replicates with triplicate technical replicates) in DNA tiling array studies relative to the WT-Y variant. Column headings: fold increase represents the change in expression and is derived from coefficient value; ID = MXAN # and description refers to annotation; both are derived from the *M. xanthus* genome data at NCBI <http://www.ncbi.nlm.nih.gov/genome/1120>.
