# Supplementary material for: Phase Variation in Myxococcus xanthus Yields Cells Specialized for Iron Sequestration
Source: PLoS One. 2014 Apr 14;9(4):e95189. doi: 10.1371/journal.pone.0095189 (PMC3986340; doi:10.1371/journal.pone.0095189)
Supplement: Table S2 — Strains and molecular reagents. (DOCX) [file pone.0095189.s004.docx]

Table S2: Strains and molecular reagents

| ***M. xanthus* strains (common name)** | | | | **Genotype** | | **Phenotype** | **Construction** | **Reference** |
| --- | --- | --- | --- | --- | --- | --- | --- | --- |
| DK1622  (WT) | | | | wild type | | Kan^S^ |  | [[1](#_ENREF_1)] |
| MxH2582  (*xre228*) | | | | ∆*xre* (MXAN_0228) | | Kan^S^ tan | Electroporated pGF120 in DK1622, screen Kan^S^ | This study |
| MxH2535  (*dkxG*) | | | | MXAN_4299::mini-Himar-GFP | | Kan^R^ tan | mini-Himar-GFP transposon into DK1622 | [[2](#_ENREF_2)] |
| MxH2602  (*asgB*) | | | | MXAN_2913::pGF143 | | Kan^R^ tan | Electroporated pGF143 into DK1622 | This study |
| ***E. coli* strains** | | | | | | | | |
| Top 10  (for cloning) | | | | F- mcrA Δ(*mrr-hsdRMS-mcrBC*) φ80l*acZ*ΔM15 Δ*lacX74 nupG* *recA1 araD139* Δ(*ara-leu*)7697 *galE15 galK16* r*psL*(Str^R^) *endA1* λ^-^ | | | | Invitrogen |
| DH5 α  (for TA assay) | | | | F^-^ *endA1 glnV44 thi-1 recA1 relA1 gyrA96 deoR nupG* Φ80d*lacZ*ΔM15 Δ(*lacZYA-argF*)U169, *hsdR*17(r_K_^-^ m_K_^+^), λ– | | | | [[3](#_ENREF_3)] |
| **Plasmid** | | | **Construction** | | | | | **Reference** |
| pCR-Blunt II-TOPO | | | cloning vector Kan^R^ , Zeo^R^ | | | | | Invitrogen |
| pGF118 | | | PCR #1 (MXAN–0227 to 0229) in pCR Blunt II TOPO | | | | | This study |
| pGF119 | | | *BlpI* digestion of pGF118 to remove *MXAN_0228* | | | | | This study |
| pGF120 | | | *EcoRI* fragment (*MXAN_0227 -0229*) from pGF118 cloned into pBJ114 | | | | | This study |
| pGF143 | | | *asgB* PCR #2 in pCR Blunt II TOPO | | | | | This study |
| **PCR products** | | |  | | | | |  |
| 1 | MXAN_0227 to MXAN_0229 | | | | | 2323 bp product; WT chromosomal DNA oligos 920x921 | | |
| 2 | MXAN_2913 fragment for disruption | | | | | 212 bp product; WT chromosomal DNA oligos with 1017x1018 | | |
| Oligonucleotides for PCR | | | | | | | | |
| # | | gene | | | oligonucleotide | | | |
| 920 | | MXAN_0227 for | | | 5’CTTCACGTAGCTCTCCTTCGGAAT | | | |
| 921 | | MXAN_0229 rev | | | 5’CATCCAACGCCAGACAAACCACTT | | | |
| 1017 | | MXAN_2913 for | | | 5’GTCGGAAGTGAAGCAGCTACAGGAG | | | |
| 1018 | | MXAN_2913 rev | | | 5’TGAGCTTGATGGACCCCGTCTCC | | | |
| Oligonucleotides for RT-PCR | | | | | | | | |
| MXAN | | | | Forward | | | Reverse | |
| 0228 (*xre)* | | | | 5' AGAAACTCGCAACCACAATCG | | | 5' CTCGGTGGCCACATCGA | |
| 3639 myxochelin | | | | 5' Caaggagttcaccaccgacaa | | | 5' caccatggccatctgcaa | |
| 3931 myxovirescin | | | | 5' Atggagggcgaccacaag | | | 5' tttcccacaacacatacacattctc | |
| 4305 (*dkx*) | | | | 5’ GACCTGGCGTACATCCTCTACAC | | | 5’ CATGGCACCACTCGATGAAG | |
| 6911 (*fepA*) | | | | 5' GACGGCAGCGTCGTCAA | | | 5' GCCGTACTCGGCGGTAATC | |
| 7370 (S_T kinase) | | | | 5' Tcaagggcaacaacatcatca | | | 5' gtcccccgagccgaagt | |

REFERENCES

1. Wall D, Kolenbrander PE, Kaiser D (1999) The *Myxococcus xanthus pilQ* (*sglA*) gene encodes a secretin homolog required for type IV pilus biogenesis, social motility, and development. J Bacteriol 181: 24-33.

2. Furusawa G, Dziewanowska K, Stone H, Settles M, Hartzell P (2011) Global analysis of phase variation in *Myxococcus xanthus*. Mol Microbiol 81: 784-804.

3. Casadaban MJ, Cohen SN (1980) Analysis of gene control signals by DNA fusion and cloning in *Escherichia coli*. J Mol Biol 138: 179-207.
